# Investigating the Effects of Valence, Arousal, Concreteness, and Humor on Words Unique to Singapore English

**DOI:** 10.5334/joc.470

**Published:** 2025-11-07

**Authors:** Cynthia S. Q. Siew, Feria Chang, Jin Jye Wong

**Affiliations:** 1 Department of Psychology, National University of Singapore, Singapore

**Keywords:** lexical-semantic norms, affective norms, valence, arousal, concreteness, humor, Singapore English, visual lexical decision

## Abstract

Singapore English is a dialect of English spoken by individuals living in Singapore, whose colloquial form (i.e., Singapore Colloquial English) contains unique lexical items not found in dominant dialects of English. The absence of these items from the lexicon of dominant English dialects indicates that lexical-semantic and affective norms central to psycholinguistic research do not exist for these Singapore English concepts, and it is unclear what is the specific influence of these effects when processing Singapore Colloquial English words. The present paper describes the development of valence, arousal, concreteness, and humor norms for a core vocabulary list of approximately 300 words and concepts, via human ratings and probing a Large Language Model, and evaluates the contribution of these norms to account for lexical processing performance in a visual lexical decision task. Results indicated that valence, arousal, and concreteness explained additional variance over and above orthographic similarity and word frequency in the visual lexical decision task. Specifically, Singapore English words that were more positively valenced, highly arousing, and more concrete, were responded to more rapidly and accurately. In addition, although there was generally a high convergence of valence, arousal, and concreteness ratings across human raters and the Large Language Model, humor norms were much less closely aligned. Overall, this paper provides a case study of how psycholinguistic research can be extended to diverse, understudied dialects of English, and showcases how doing so offers an opportunity for psycholinguistics to examine the importance of various lexical-semantic and affective measures to quantify lexical information in colloquial, informal language.

## Introduction

A core activity of psycholinguistic research revolves around the development of large-scale lexical-semantic and affective norms for lexical items in various languages. Lexical norms broadly refer to a set of lexical, affective, and semantic properties that meaningfully characterize lexical items that make up the lexicon of a language. Well known norm databases include Warriner et al. (2013)’s valence, arousal, and dominance ratings for over 13,000 English words and Brysbaert, Warriner, et al. (2014)’s concreteness ratings for 40,000 English words. More recently, researchers have also developed lexical norms of socialness (Diveica et al., 2022) and sensorimotor norms quantifying perceptual and action strength (Lynott et al., 2020). Evidently, these measures capture meaningful dimensions of the semantics of words and concepts, which have important implications for lexical processing (Diveica et al., 2024).

The aims of this paper are as follows: First, we present lexical-semantic and affective norms for words and concepts unique to Singapore English, the dialect of English spoken by individuals who reside in Singapore. Second, we show that these norms have detectable effects on the lexical processing of words unique to Singapore English in a visual lexical decision task. It is important to emphasize that these words and concepts (e.g., *shiok, makan, see me no up*) are unique to Singapore English, and do not exist in current databases of English word norms. Therefore, even though these lexical items are well known and commonly used among speakers of Singapore English (Siew, 2024b), basic information about their valence, arousal, concreteness, and humor, is lacking from the scientific record. Our goal is to fill this gap by developing lexical-semantic and affective norms for these items, and to showcase their effects in a classic psycholinguistic task, which we hope will enable and motivate future psycholinguistic research on Singapore English that includes these lexical items, rather than excludes them.

### Introduction to Singapore English

Singapore English is the dialect of English spoken by individuals who reside in Singapore, a country in Southeast Asia which was formerly a British colony (see Bolton et al. (2020), for an overview of Asian Englishes and how they are influenced and shaped by British colonization in the region). Sociolinguists who study Singapore English recognize that it is comprised of two varieties that sit at two ends of a continuum in a diglossic context (Gupta, 1994): Singapore Standard English (SSE) and Singapore Colloquial English (SCE), also commonly known as “Singlish” in popular culture. Standard Singapore English is the “high” or prestige variety used in written and formal contexts, and is not particularly different from other dominant varieties of English such as British English and North American English. On the other hand, Singapore Colloquial English is the “low” variety commonly used in informal contexts (Cavallaro et al., 2020; Ching, 2020; Tan, 2017), and is generally perceived as a “broken”, improper form of English that potentially threatens the country’s economic progress and global standing (Rubdy, 2001).

In this paper, we were particularly interested in the colloquial form of Singapore English. This is because SCE consists of several lexical items and lexical borrowings from other languages that do not necessarily have an equivalent form in SSE. An example is the word *paiseh*, which is derived from the Chinese dialect Hokkien, and refers to feeling embarrassed and is also used in place of “excuse me” or “sorry” (as an interjection). It is also noteworthy that given the creole status of Singapore English these concepts are initially and predominantly used in spoken communication among various ethnic groups residing in Singapore. With the advent of new technologies such as email, text messaging, the Internet, and social media, many of these spoken forms acquired written forms and are still commonly used among speakers of Singapore English in both online and face-to-face communication (Gonzales et al., 2023). Although there has been previous efforts leveraging distributional semantics and machine learning approaches to infer sentiment of SCE vocabulary (Ho et al., 2018; Lo & Lim, 2018), validated lexical norms and psycholinguistic repositories of Singapore Colloquial English vocabulary developed using behavioral data collected from users of the languages themselves are lacking and not particularly well established.

### Limited linguistic coverage in language research

It is well established that research in the language and cognitive sciences is dominated by the study of English speakers (Blasi et al., 2022), and in particular, by speakers of dominant, prestigious dialects of English, such as Standard North American English and British English (Levisen, 2019). When we narrow our focus on the languages that are represented in lexical-semantic databases or psycholinguistic megastudies, the same pattern of dominance by a handful of languages is also observed. Siew (2024a) conducted a brief analysis of languages represented in linguistic databases listed in the Linguistic Annotated Bibliography (Buchanan et al., 2019) and found that approximately 53% of all databases were in English, with only 8 other languages (Spanish, German, French, Chinese, Italian, Dutch, Portuguese, Japanese) comprising almost a third of all databases. Moreover, a large majority of databases involved participants who spoke American or British English. A notable exception is a prominent megastudy of auditory lexical decision which was developed with data from Singapore English speakers (Goh et al., 2020); however, all the words in their database consisted of words found in standard dialects of English and do not include any of the items that we study in this paper.

Despite greater awareness of the limited linguistic coverage in language research, it is striking that the situation has not improved much based on recent bibliometric reviews (Kidd & Garcia, 2022; Singh et al., 2023; Thalmayer et al., 2021). We hope that conducting the research detailed in the present paper can provide a small but meaningful contribution to psycholinguistic research, and showcase how studying colloquial language can offer new opportunities and raise interesting research questions for psycholinguistics.

### Lexical-semantic and affective norms: Valence, arousal, concreteness, and humor

In the present paper, we developed lexical-semantic and affective norms of valence, arousal, concreteness, and humor. Although there are many possible norms that could have been established (e.g., sensorimotor ratings or socialness), we decided to focus on these four as an initial starting point and because these are considered to be highly central and important norms, as evidenced by the extensive literature where these norms have been established for thousands of words and concepts in English (i.e., high lexicon coverage; e.g., (Brysbaert, Warriner, et al., 2014; Engelthaler & Hills, 2018; Warriner et al., 2013)) and in languages other than English (e.g., valence and arousal norms for Spanish (Stadthagen-Gonzalez et al., 2017), concreteness norms for Dutch (Brysbaert, Stevens, et al., 2014)). Below we briefly discuss what these norms are and their relevance for psycholinguistic studies.

Valence and arousal are affective ratings of the emotions elicited by a particular word or concept (Osgood et al., 1957). Valence refers to the pleasantness of the emotion elicited, ranging from highly pleasant (e.g., *joke* and *kitten*) to unpleasant (e.g., *jail* and *kill*). Arousal refers to the degree of arousal invoked, ranging from highly arousing (e.g., *sex* and *thrill*) to unexciting (e.g., *statue* and *bored*). The development of these affective norms support research on the contributions of emotional features to memory and lexical processing (Kousta et al., 2009; Kuperman et al., 2014) and for sentiment analyses of written text (Fatima et al., 2021; Mohammad & Turney, 2013; Teixeira et al., 2021). The psycholinguistic literature generally reports a facilitatory effect for positive words (Haro et al., 2024) or a facilitatory effect for emotionality, where words that are positively and negatively valenced experience a processing advantage relative to neutral words (Kousta et al., 2009). As for arousal, the general pattern is that highly arousing words are recognized less slowly (Kuperman et al., 2014), in line with the automatic vigilance model of emotion (Pratto & John, 1991), where highly arousing and negatively-valenced stimuli automatically capture attentional resources which slows processing.

Concreteness refers to the extent to which the concept represented by a word (or short phrase) refers to a perceptible entity (Brysbaert, Warriner, et al., 2014; Paivio, 2013). In accordance with Paivio’s Dual Coding Theory, highly concrete words are easier to remember than more abstract words because they activate both perceptual and verbal codes in memory, whereas abstract words only activate a verbal code (Paivio, 2013). An alterative explanation for the concreteness effect is offered by the Context Availability Theory (Schwanenflugel et al., 1988), where concrete words benefit from being readily associated with rich contextual information relative to abstract words. In this study, words that receive a higher concreteness rating refers to an entity that exists in the real world and elicits immediate experiences of that concept through one’s senses. Words that receive a lower concreteness rating are considered “abstract” and refers to an entity that cannot be directly experienced through one’s senses. Concreteness is another important construct for psycholinguistic and memory research, as evident from studies that have shown how concreteness/abstractness influences various cognitive and lexical process such as word recognition (Goh et al., 2016), word learning (Groot & Keijzer, 2000), humor perception (Siew et al., 2022; Westbury & Hollis, 2019), and historical language change and evolution (Hills & Adelman, 2015; Y. Li et al., 2024). Specifically, the facilitative effects of concreteness is well-established within the domains of lexical processing and word recognition, where concrete words are responded to more quickly and accurately as compared to abstract words (Bottini et al., 2022; Goh et al., 2016).

Finally, humor norms capture the extent to which a word elicited feelings of amusement or humorous thought (Engelthaler & Hills, 2018). Examples of humorous English words include *booty* and *waddle*, and examples of the least humorous words include *nightmare* and *pain*. To the best of our knowledge, although there has been some interest in quantifying the humor of nonwords (Westbury et al., 2016) and of word pairs (Siew et al., 2022; Westbury & Hollis, 2019), there is only one existing database of word humor norms in English (Engelthaler & Hills, 2018). In that paper, the authors found that word humor was associated with several other lexical-semantic and affective variables such as concreteness and valence, but was most strongly anti-correlated with word frequency, such that humorous words tend to be more rare in language. Word humor was also positively correlated with lexical decision RTs obtained from the English Lexicon Project, such that humorous words were more slowly responded to in visual lexical decision. Although humor is a somewhat more recent addition to the realm of psycholinguistic norms, we decided to collect humor ratings for our items because research has demonstrated interesting connections between the use of colloquial language to elicit humor (Joser et al., 2023; Ruiz-Gurillo, 2021) and we anticipate that the collection of humor norms for SCE could enable future investigations into the socio-pragmatic effects of colloquial language use in a Singaporean context.

Finally, another key aspect of the current paper is to also collect visual lexical decision data for SCE words, so that we can evaluate the ability of these lexical-semantic and affective norms in explaining lexical processing performance. The visual lexical decision task is a classic psycholinguistic task that is commonly used to explore the influence of various lexical variables on lexical retrieval. Here, we were interested to see if valence, arousal, concreteness, and humor would show similar patterns and effects in the recognition of SCE lexical items as reported in the prior literature. Based on the discussion above, we expected to find facilitative effects for SCE words of higher concreteness, positive valence, low arousal, and low humor.

### Why do we need to develop lexical norms for Singapore English?

As mentioned earlier, Singapore English is one of the many dialects of English that exist worldwide. Given that English serves as a lexifier for SCE, it is unsurprising to find a large overlap of vocabulary among Singapore English and other English dialects. However, unpublished data from our lab suggests that there are approximately 4,000 words, phrases, and concepts that are unique to Singapore. We would like to emphasize that the present investigation is focused on lexical items *unique* to SCE and thus not found in the vocabulary of other major English dialects. Specifically, among the list of 283 items that we collected lexical-semantic and affective measures for in this paper, only 9 of these items were found in the English Lexicon Project, a megastudy of North American English (Balota et al., 2007) and only 12 were found in the British Lexicon Project, a megastudy of British English (Van Heuven et al., 2014). Therefore, lexical-semantic and affective information and behavioral data was only available for a small proportion of the items in our current study. This also necessitated the need to collect new visual lexical decision data (Part 3) for validation, as opposed to simply re-analyzing pre-existing megastudy datasets.

### Leveraging Large Language Models for Developing Lexical Norms

There has been recent interest among psycholinguists to harness the capabilities of Large Language Models (LLMs) to develop lexical-semantic and affective norms for psycholinguistic research at a massive scale (Trott, 2024). Some recent papers have suggested that this is a promising avenue, showing that LLMs are able to generate valid, high-quality lexical-semantic and affective norms such as familiarity, valence, arousal, and concreteness, for English words and multi-word expressions (Martínez, Molero, et al., 2024) and Spanish words (Martínez, Conde, et al., 2024) at a fraction of the cost and time that is usually invested in human-based data collection. The results for Spanish are particularly interesting as they suggest that even if the LLM is not predominantly trained on the target language, it is still able to produce reasonable estimates (Martínez, Conde, et al., 2024). Hence, a secondary goal of this paper to explore the potential of LLMs to provide lexical-semantic and affective estimates of concepts unique to a minority English dialect, Singapore English, and to compare its explanatory power in a behavioral task to traditionally obtained norms. If AI-generated norms are comparable to human ratings and produce expected behavioral results when used as predictors, perhaps LLMs could be leveraged to produce lexical norms for under-resourced languages and dialects in future work.

### Outline of paper

We first describe the development of lexical-semantic and affective norms for a subset of items in the SCE vocabulary. In Part 1 we show that participants are able to provide fairly reliable ratings for these items, and Part 2 describes the process by which the same ratings were obtained from GPT-4. We then report the results of a visual lexical decision (VLD) study to assess if these lexical-semantic and affective measures have any measurable impact on lexical processing in Part 3. We demonstrated that some of these measures are able to account for additional variance in VLD performance beyond traditional measures such as word length and frequency.

## Part 1: Development of lexical-semantic and affective norms

This section describes the data collection and analysis process involved in developing lexical-semantic and affective norms for a set of Singapore English concepts.

### Method

#### Participants

A total of 360 participants were recruited from the National University of Singapore (NUS) Psychology Undergraduate Research Pool. Participants were required to be native speakers of Singapore English and received credit that went towards fulfilling a course requirement. The study was approved by the Institutional Review Board (NUS-IRB-2022-886: *Word associations and lexical-semantic norms for Singapore English*) at the National University of Singapore.

After exclusions (see Results), our final sample consisted of 297 participants: 90 provided valence ratings, 82 provided arousal ratings, 63 provided concreteness ratings, and 62 provided humor ratings. Each participant rated all 283 items, but only on a single dimension. Out of the final sample of 297 participants, there were 198 female participants and 99 male participants. The mean age was 20.8 years (*SD* = 2.1). 208 participants identified as Chinese, 11 identified as Malay, 6 identified as Indian, and 9 identified as other ethnicities; ethnicity information was not available for the 63 participants in the Concreteness group.

#### Stimuli and Study Materials

The stimuli consisted of 283 words and short phrases selected from the Wikipedia page on Singlish vocabulary (https://en.wikipedia.org/wiki/Singlishvocabulary; last accessed 4^th^ January 2024). This set of stimuli is part of a more substantial list of lexical borrowings and unique concepts in Singapore English which is manually curated by our research group. A few examples of the stimuli include: “zha bor” (noun, meaning: a young girl), “suay” (adjective, meaning: unlucky), and “sakar” (verb, meaning: to flatter). Overall, the stimuli consist of a wide range of grammatical types (the top categories are verbs [35.0%], nouns [32.2%], adjectives [30.7%], interjections [14.5%], and adverbs [5.3%]; note that these do not add up to 100% as some words have multiple roles) and with different etymologies or origin languages (the top categories are Hokkien [48.4%], Malay [23.0%], English [18.7%], and Cantonese [7.8%]). A complete list of the stimuli and their lexical information can be found on the OSF and on this website: http://r-server.csqsiew.xyz/sgnorms/. We decided to begin collecting norms for these concepts because these were concepts that were likely to be prominent, central concepts in the lexicon of Singaporean English speakers based on our previous work (Siew, 2024b; Wong & Siew, 2024).

We also selected a number of calibrator items for each word rating task. Calibrator words were English words selected from original word norm databases to provide examples of the extreme ends and mid-point of the scale. These words were shown at the beginning of the study in order to calibrate the participant to the full range of the scale prior to the rating of the Singapore English words. For instance, “grasshopper” (4.91) and “theory” (1.47) served as the high and low calibrator items for concreteness, and “marriage” (2.51) served as the mid-point, with their original ratings in parentheses. The full list of calibrator words can be found on our OSF.

#### Procedure

First, participants were provided with a participant information sheet explaining the goals and requirements of the study. They provided informed consent before proceeding. Participants then completed the word rating task, followed by a short demographic survey, and were debriefed on the purpose of the study. Participants were presented with the list of calibrator words first, followed by the main list of 283 Singapore English words and short phrases. The order of presentation within the main list was randomized across each participant.

We attempted to align our data collection procedure to be as close as possible to that of prominent papers which developed word norms in English. Hence, for valence and arousal, a 9-point Likert rating scale was used in accordance with Warriner et al. (2013), whereas for concreteness and humor, a 5-point Likert rating scale was used in accordance with Engelthaler and Hills (2018) and Brysbaert, Warriner, et al. (2014).

Participants were instructed that they would be shown a list of words and short phrases that were unique to Singapore English, and their task was to rate how they felt while reading each word or phrase. The rating scale ranged from 1 (most negatively valenced; least arousing; most abstract; most humorless) to 5 or 9 (most positively valenced; most arousing; most concrete; most humorous). Participants were also informed to select a specific option on the screen if they did not know the word or phrase well enough to provide a rating. Specific instructions for each rating task can be found in our OSF.

### Results

Data exclusion followed a two-tiered approach. In the first step, we excluded participants who self-reported that English was not their native/first language, that they had a prior history of speech or language disorders, or that they were significantly distracted while completing the task. We also excluded participants who did not consider themselves current residents of Singapore (e.g., exchange students), or had resided in Singapore for less than 10 years. Out of the initial 360 participants, 324 participants remained after applying these criteria. In the second step, we excluded participants who provided low-quality data as determined by lack of variability in responses (i.e., the same rating was provided to all words; *n* = 4), poor correlation of ratings (less than 0.1) with the average ratings of the other raters (*n* = 20), or poor lexical knowledge of a high proportion of the items presented (*n* = 3). Specifically, participants who gave the same response to all items, or whose own ratings correlated poorly with the overall mean ratings, or who indicated that they did not know more than 60% of the items presented, were excluded from data analysis. These additional data cleaning steps are typically done in other studies that developed lexical-semantic word norms (Brysbaert, Warriner, et al., 2014).

Then, trials where the participants indicated that they did not know the word or phrase were also removed. Using the remaining trials, valence, arousal, concreteness, and humor norms were computed for each item using cumulative link mixed effect modeling to obtain latent norms (Taylor et al., 2022). Although item norms are commonly computed by taking the average of the rating for each word, Taylor et al. (2022) showed that the use of cumulative link mixed-effects models (CLMM) to compute item norms has several advantages, including taking into account the ordinal nature of Likert ratings (instead of treating ratings as continuous), and being less influenced by response biases of the participants. Specifically, the CLMM models Likert scale ratings as ordinal dependent variables which are mapped onto ordered regions of a normally distributed latent distribution, and since these CLMMs are fitted with item random effects, the extracted random effects of the model represents estimate of the latent mean for each item (i.e., the item’s latent norm on a particular dimension) (Taylor et al., 2022).

Although lexical norms are analyzed using the CLMM approach in this manuscript, traditionally computed item norms are available in the OSF for interested readers to review. The trial-level data was submitted to a R Shiny application developed by Taylor et al. (2022) (see: https://github.com/JackEdTaylor/shinynorms) to compute latent word norms using cumulative link mixed-effects models with a probit link function. All data (trial-level rating data, lexical norms, stimuli) and analysis scripts can be found at https://osf.io/bjxfa.

**Figure 1:**
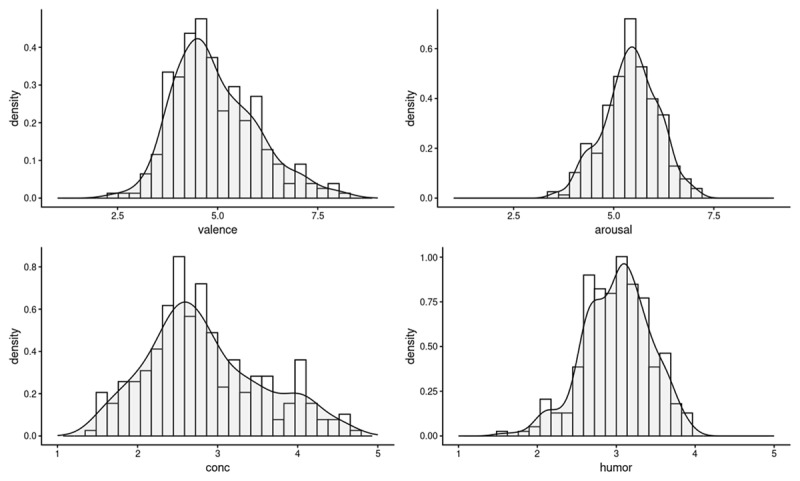
Density plots for valence, arousal, concreteness, and humor ratings provided by human raters.

Table 1 shows examples of items that received the highest and lowest ratings on each of the four measures, and Table 2 shows a correlation table of the four measures collected for 283 items. Figure 1 shows the distributions for each of the four norms, and Table 3 presents basic descriptive information for their distributions.

**Table 1:** Examples of Singapore English Concepts.


HIGHEST			LOWEST		
	
WORD	M	SD	WORD	M	SD

**Valence**					

ang bao/hong bao	8.29	1.07	chee ko pek/ti ko pek	2.33	1.60

huat	8.00	1.31	Ah Long	2.61	1.52

shiok	7.88	1.43	saman	2.80	1.54

makan	7.87	1.42	O$P$	3.11	2.16

pasar malam	7.54	1.26	hao lian	3.14	1.55

**Arousal**					

bo ta bo lan pa	7.12	1.58	lepak	3.51	2.18

chee bai	7.01	1.78	meh	3.59	1.92

huat	6.99	1.84	abit	3.69	1.82

si mi lan jiao	6.85	1.91	helication	3.92	1.73

hong gan	6.83	1.83	handphone	4.01	1.97

**Concreteness**					

kopi	4.68	0.78	mah	1.41	0.74

handphone	4.65	0.83	ah	1.52	0.97

ang bao/hong bao	4.56	0.88	lah	1.52	0.94

kopitiam	4.52	1.00	ar	1.53	0.88

makan	4.52	0.90	nia	1.56	0.86

**Humor**					

your head	3.95	0.97	handphone	1.56	0.85

pak chiu cheng	3.89	1.18	kopitiam	1.77	0.88

sod	3.86	0.88	kampung	1.93	0.91

bo geh	3.85	1.10	da bao	2.00	0.98

piak piak	3.84	1.27	kopi	2.05	1.03


**Table 2:** Correlation of lexical-semantic and affective measures for 282 Singapore English items.


	VALENCE	AROUSAL	CONCRETENESS	HUMOR

Valence	1	–0.34***	0.12*	–0.41***

Arousal	–0.34***	1	–0.01	0.55***

Concreteness	0.12*	–0.01	1	–0.26***

Humor	–0.41***	0.55***	–0.26***	1


* *p* < .05, *** *p* < .001.

**Table 3:** Descriptive information for raw human ratings.


RATING	MEAN	SD	MEDIAN	MIN	MAX	RANGE	SKEW	KURTOSIS

Valence	4.94	1.04	4.75	2.33	8.29	5.96	0.62	0.26

Arousal	5.42	0.68	5.45	3.51	7.12	3.61	–0.19	–0.18

Concreteness	2.83	0.73	2.72	1.41	4.68	3.27	0.47	–0.38

Humor	3.00	0.42	3.03	1.56	3.95	2.39	–0.35	0.13


#### Assessing reliability

To evaluate the ratings’ internal reliability, split-half correlations were calculated separately for each measure. For each measure, participants were divided into two groups of even- and odd-numbered participants, and their ratings were used to compute latent item norms for all items. Ratings from the odd and even groups were found to correlate very highly across all four measures (valence: *r*(281) = 0.92, *p* < .001; arousal: *r*(281) = 0.84, *p* < .001; concreteness: *r*(281) = 0.91, *p* < .001; humor: *r*(281) = 0.91, *p* < .001).

Intra-class correlation coefficients (ICCs) were also calculated for each measure separately using average measures, two-way random effects model, and absolute agreement (Bartko, 1966; Shrout & Fleiss, 1979). An ICC was obtained for each item (i.e., word) rated by the participants and the mean ICC was calculated by averaging the ICCs of all the items. The mean and standard deviation of the ICCs were generally high: valence: 0.973 (95% *CI* [0.96, 0.983]; *F*(45, 2279) = 42.0, *p* < .001), arousal: 0.903 (95% *CI* [0.862, 0.937]; *F*(50, 1080) = 13.2, *p* < .001), concreteness: 0.97 (95% *CI* [0.958, 0.98]; *F*(54, 1445) = 39.7, *p* < .001), humor: 0.913 (95% *CI* [0.881, 0.939]; *F*(77, 492) = 16.8, *p* < .001). *F*-tests indicated that the ICCs significantly differed from the null hypothesis of 0. Overall, high ICCs were obtained for all four measures, indicating high inter-rater reliability and consistency in the sample.

## Part 2: AI-generated estimates of Singapore English lexical-semantic and affective norms

This section describes the data collection and analysis process involved in generating lexical norms for a set of Singapore English concepts from a LLM, to explore the possibility of using LLMs to complement human-based data collection for the development of word norms.

### Method

Our approach closely followed that of Martinez and colleagues (Martínez, Conde, et al., 2024; Martínez, Molero, et al., 2024). To query GPT-4 about various lexical-semantic and affective features of the SCE word or concept, we provided it with two prompts that was adapted from Martínez, Molero, et al. (2024). We used the MacBehaviour R package (Duan et al., 2024) to automate the interactions with GPT-4 (gpt-4o-2024-11-20). Although there are many other LLMs that could have been queried, we focused on GPT-4 since it was previously shown to provide the best results (Martínez, Conde, et al., 2024; Martínez, Molero, et al., 2024). We provide an example of the specific prompt used for *valence* below (a complete list of prompts used can be found in the OSF):

Could you please rate how reading the following word/multi-word expression makes a person feel. Use a scale from 1 to 9, where 1 means very negative, bad and 9 means very positive, good. Examples of words that would get a rating of 1 are pedophile, AIDS, and wreck. Examples of words that would get a rating of 9 are vacation, fantastic, and laugh. The word/multi-word expression is: [WORD/MWE]. Only answer a number from 1 to 9. Please limit your answer to numbers.

To explore the possibility that we might get better results by “nudging” the model into a Singapore English context, we also queried the model with a second slightly modified prompt. An example for *valence* shown below, with differences from the previous prompt highlighted in bold:

Could you please rate how reading the following **Singapore English** word/multi-word expression makes a person feel. Use a scale from 1 to 9, where 1 means very negative, bad and 9 means very positive, good. Examples of words that would get a rating of 1 are pedophile, AIDS, and wreck. Examples of words that would get a rating of 9 are vacation, fantastic, and laugh. The **Singapore English** word/multi-word expression is: [WORD/MWE]. Only answer a number from 1 to 9. Please limit your answer to numbers.

The temperature parameter in LLMs influences the randomness of generated responses. In this case, the temperature of the model was set to 0, so that the same results (i.e., least random) are obtained each time a word is presented to the model. We also extracted the estimated probabilities of the 5 most likely answers (via the “log_probs” function). For instance, if the word *shiok* received a probability of .6 (rounded off) for rating 7 (high valence), .3 for rating 6, .05 for rating 8, and .025 for ratings 5 and 9, one could calculate two values: the raw rating with the highest probability (i.e., 7) and a more precise, weighted value found by multiplying the ratings with their probabilities. For *shiok* this would give a value of .6 × 7 + .4 × 6 + .05 × 8 + .025 × 5 + .025 × 9 = 7.35. Finally, the prompt was repeated anew for each item to prevent instruction dilution.

To summarize, for each of the 283 SCE concepts, we obtained both a raw and weighted numerical rating for valence, arousal, concreteness, and humor from GPT-4. This was repeated twice, the first set of ratings was obtained from a general prompt with no reference to Singapore English, and the second set of ratings was obtained from a specific prompt with an explicit reference to Singapore English.

### Results

To evaluate the quality of the LLM-generated ratings, we correlated the four numeric ratings obtained from GPT-4 (raw/weighted rating × general/specific prompt) with the human-generated ratings described in Part 1. Table 4 shows the Pearson correlations among these measures. All correlations were statistically significant at the *p* < .01 level.

**Table 4:** Correlations of human ratings with ChatGPT ratings.


MEASURE	GENERAL (RAW)	GENERAL (WEIGHTED)	SPECIFIC (RAW)	SPECIFIC (WEIGHTED)

Valence	0.40	0.42	0.76	0.78

Arousal	0.26	0.27	0.57	0.59

Concreteness	0.29	0.31	0.66	0.69

Humor	0.18	0.19	0.33	0.39


All correlations were statistically significant, all ps < .01.

**Figure 2:**
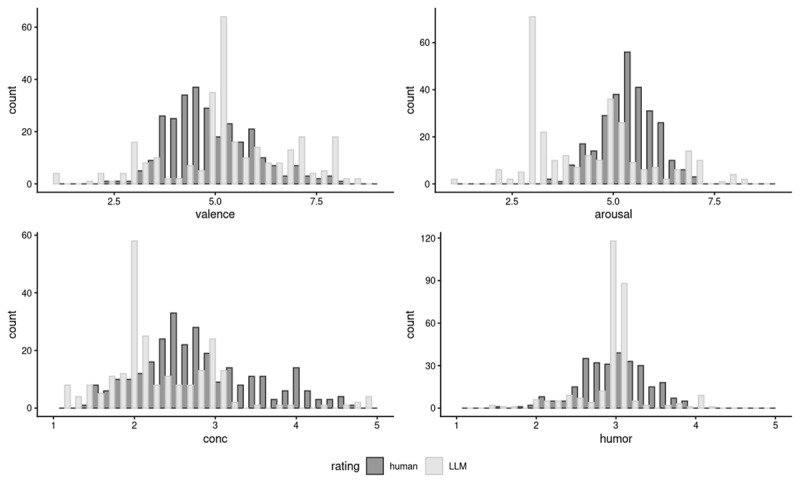
Density plots for valence, arousal, concreteness, and humor ratings generated by GPT4 (specific, weighted condition) overlaid on the corresponding human ratings.

Figure 2 shows the distributions for each of the four norms generated by GPT and the same norms provided by human raters, to provide a visual comparison. Table 5 presents basic descriptive information of the LLM distributions. A 2-sample Kolmogorov-Smirnov non-parametric test was used to determine whether human-generated and LLM-generated distributions differed significantly across each of the four norms. The results indicated that the human-generated and LLM-generated distributions were significantly different; valence: *D* = 0.29, arousal: *D* = 0.47, concreteness: *D* = 0.41, humor: *D* = 0.40, all *p*s < .001.

**Table 5:** Descriptive information for LLM ratings (specific, weighted).


RATING	MEAN	SD	MEDIAN	MIN	MAX	RANGE	SKEW	KURTOSIS

Valence	5.30	1.55	5.08	1.00	9.00	8.00	–0.19	–0.01

Arousal	4.36	1.45	4.33	1.03	8.07	7.04	0.51	–0.50

Concreteness	2.40	1.09	2.06	1.00	5.00	4.00	1.12	0.70

Humor	2.93	0.40	3.00	1.00	4.17	3.17	–0.78	5.06


There are a few interesting results in the correlation analyses. First, correlations of human ratings and GPT ratings obtained with a general prompt were smaller than the correlations with GPT ratings obtained with a specific prompt, across all four measures. This points to the importance of carefully designing prompts to focus the model’s output in a specific context, in this case, Singapore English. Second, correlations were slightly higher for the weighted ratings as compared to the raw ratings, in accordance with previous research (Martínez, Molero, et al., 2024). This indicates that obtaining a weighted value based on estimated probabilities can be one way of improving the quality of LLM-generated ratings, as compared to simply taking the first answer (i.e., raw rating). Third, correlations were highest for valence, followed by concreteness, arousal, and humor. This also appears to be consistent with (Martínez, Conde, et al., 2024; Martínez, Molero, et al., 2024) who found the highest correlations for valence than arousal in both Spanish and multi-word expressions in English. It appears that LLM performance is quite poor for humor. Finally, the best performing measure (weighted ratings + specific prompt) produced estimates that correlated well with human ratings, albeit these values were on the lower end of the range of correlations that have been reported in the literature. In the next section, we will evaluate the performance of these LLM-generated measures to account for human performance in a visual lexical decision task.

## Part 3: Visual lexical decision task

To determine if the norms collected for Singapore English concepts had any observable implications for lexical processing, a visual lexical decision task was conducted using a subset of items. We expected to find that lexical-semantic and affective norms would explain additional variance in lexical decision performance beyond that of word frequency and other orthographic variables.

### Method

#### Participants

A total of 74 participants were recruited from the NUS Psychology Undergraduate Research Pool. Participants were required to be speakers of Singapore English and received credit that went towards fulfilling a course requirement. The study was approved by the Institutional Review Board (NUS-IRB-2022-886: *Word associations and lexical-semantic norms for Singapore English*) at the National University of Singapore.

Data exclusion followed a two-tiered approach. In the first step, we excluded participants who self-reported that English was not their native/first language, that they had a prior history of speech or language disorders, or that they were significantly distracted while completing the task. 58 participants remained after applying these criteria. In the second step, we excluded 2 participants whose overall accuracy rate was very low (*n* = 1) and overall mean reaction time was very slow (*n* = 1) relative to the rest of the participants (i.e., more than 2.5 *SD*s from the group mean).

Our final sample consisted of 56 participants. There were 31 female, 23 male, and 2 participants who did not wish to disclose their gender. The mean age was 21.6 years (*SD* = 2.4). 48 participants identified as Chinese, 3 identified as Malay, 1 identified as Indian, and 4 identified as Mixed/Others.

#### Stimuli and Study Materials

Out of the list of 283 words and short phrases for which lexical norms were collected for in Part 1, 136 items were selected as word stimuli in the visual lexical decision task. Short phrases were excluded from the selection process such that only single words could be selected as stimuli. Due to a spelling error, one of the 136 words was subsequently excluded from subsequent analyses. 136 nonwords were created with the UniPseudo pseudoword generator (New et al., 2024), which uses an algorithm based on Markov chains of orthographic n-grams to ensure that nonwords have similar orthographic characteristics as the words.

The following variables were also obtained for the word stimuli for inclusion as covariates in the subsequent analyses: number of letters, orthographic neighborhood size, mean bigram frequency, and word frequency. Orthographic neighborhood size refers to the number of orthographic neighbors a word has based on the 1-edit distance metric. Mean bigram frequency refers to the average bigram count for a particular word. These two measures were obtained from the English Lexicon Project web application (Balota et al., 2007) (https://elexicon.wustl.edu). Word frequency measures were obtained from the National Speech Corpus, a large spoken corpora of Singapore English (Koh et al., 2019) (https://www.imda.gov.sg/how-we-can-help/national-speech-corpus). In accordance with Brysbaert and Diependaele (2013)’s suggestion, words missing from the corpora (i.e., have zero frequency) were submitted to a Laplace transformation whereby 1 is added to the frequency count and the corpus size is increased by the number of word types. Zipf values were then computed for the word items in accordance with the equations in Van Heuven et al. (2014) and these were used as our word frequency measure in the subsequent analyses.

A full list of words and nonwords used in the visual lexical decision can be found on the OSF page. Table 6 shows the summary descriptives of the lexical and semantic measures associated with the word stimuli and Table 7 shows the correlations among the same variables.

**Table 6:** Descriptive statistics for 135 word items.


STATISTIC	N	MEAN	ST. DEV.	MIN	MAX

no. of letters	135	4.87	1.67	2	12

orthographic neighborhood size	135	4.01	5.74	0	26

mean bigram frequency	135	2,907.95	1,485.98	226.00	6,993.67

log frequency	135	2.80	1.60	0.00	6.76

valence	135	0.18	0.61	–1.26	1.90

arousal	135	–0.13	0.36	–1.00	1.00

concreteness	135	–0.02	0.74	–1.51	1.92

humor	135	–0.13	0.43	–1.64	0.80


**Table 7:** Correlation of lexical-semantic and affective measures for 135 word items.


	NO. OF LETTERS	ORTHOGRAPHIC NEIGHBORHOOD SIZE	MEAN BIGRAM FREQUENCY	LOG FREQUENCY	VALENCE	AROUSAL	CONCRETENESS	HUMOR

no. of letters	1	–0.62***	0.21*	–0.44***	–0.11	–0.03	0.30***	0.06

orthographic neighborhood size	–0.62***	1	0.07	0.39***	0.10	–0.12	–0.29***	–0.04

mean bigram frequency	0.21*	0.07	1	–0.03	–0.02	–0.08	0.11	–0.01

log frequency	–0.44***	0.39***	–0.03	1	0.35***	–0.18*	–0.10	–0.29***

valence	–0.11	0.10	–0.02	0.35***	1	–0.28***	0.08	–0.34***

arousal	–0.03	–0.12	–0.08	–0.18	–0.28***	1	0.001	0.52***

concreteness	0.30***	–0.29***	0.11	–0.10	0.08	0.001	1	–0.25**

humor	0.06	–0.04	–0.01	–0.29	–0.34***	0.52***	–0.25**	1


* *p* < .05, ** *p* < .01, *** *p* < .001.

#### Procedure

Participants were first presented with information about the study before providing informed consent to proceed with the visual lexical decision task. In each trial, a fixation cross appeared on the screen for 500 ms. A letter string appeared in the center of the screen in uppercase. Participants were instructed to decide, as quickly and accurately as possible, whether the letter string formed a real word in Singapore English or a nonword. If the item was a word, participants pressed the ‘J’ button on the keyboard with their right index finger. If the item was a nonword, participants pressed the ‘F’ button with their left index finger. Reaction times (RTs) were measured from stimulus onset to the onset of the participant’s button press. The next trial began 500 ms after the participant’s response was made. If no response was made within 3s, the next trial automatically began. Prior to the experimental trials, each participant received ten practice trials to become familiar with the task; these trials were not included in the subsequent analyses. The presentation order of the words and nonwords were randomized for each participant, and the trials were divided into four separate blocks with a short break in between. After completing the visual lexical decision task, participants completed a short demographic survey and were debriefed.

### Results

For the RT data, errors and nonword trials were first excluded. Trials with latencies that were 2.5 *SD*s above or below each participant’s mean RT were considered outliers and excluded from analysis. This resulted in 3.1% of the data being removed.

Using the *lme4* R package, a linear mixed effects (LME) model was used to predict RTs and a generalized linear mixed effects (GLM) model was used to predict accuracy from the lexical decision data (Bates et al., 2014). Both RT and Accuracy models included the following predictors: (a) random intercept effects of participants and items, (b) fixed effects of interest (valence, arousal, concreteness, humor), and (c) fixed effects for control variables (number of letters, word frequency, orthographic neighborhood size, and mean bigram frequency). All predictor variables were standardized. The Satterthwaite’s method as implemented in the *lmerTest* R package (Kuznetsova et al., 2017) was used to obtain the degrees of freedom and *p*-values for all the linear mixed effects models reported in this paper.

A two-step hierarchical approach was used. Number of letters, word frequency, orthographic neighborhood size, mean bigram frequency were entered into the LME model in Step 1 (base model). Valence, arousal, concreteness, and humor were entered into the LME model in Step 2 (base+norms model), in addition to the previously entered variables. Partitioning the analysis into two steps was done to determine if the lexical-semantic and affective measures collected in the previous section accounted for additional variance over conventional lexical variables.

#### RT

Table 8 presents the results of the LME model for RTs. The overall mean RT was 916 ms (*SD* = 175 ms). The following fixed effects were significant: number of letters, orthographic neighborhood size, word frequency, valence, arousal, and concreteness. Three out of the four variables of interest were significant predictors of RT. Specifically, positively valenced words were responded to more quickly than negatively valenced words (standardized *β* = –20.3, *SE* = 10.0, *t* = –2.03, *p* = .045), highly arousing words were responded to more quickly than less arousing words (standardized *β* = –27.7, *SE* = 11.4, *t* = –2.44, *p* = .016), and concrete words were responded to more quickly than abstract words (standardized *β* = –24.4, *SE* = 10.4, *t* = –2.35, *p* = .021). Humor did not significantly predict RTs. The likelihood ratio test indicated that the inclusion of lexical-semantic and affective measures significantly improved RT model fit, *χ*^2^ = 19.8, *df* = 4, *p* < .001.

**Table 8:** Visual Lexical Decision Models.


	*DEPENDENT VARIABLE*			

	RT		ACC	
	
	*LINEAR MIXED-EFFECTS*		*GENERALIZED LINEAR MIXED-EFFECTS*	
	
	BASE	BASE+NORMS	BASE	BASE+NORMS
	
	(1)	(2)	(3)	(4)

no. of letters	54.121*** (11.434)	52.229*** (11.246)	0.006 (0.170)	0.041 (0.156)

orthographic neighborhood size	64.943*** (12.387)	56.943*** (12.024)	–0.473** (0.167)	–0.397** (0.152)

mean bigram frequency	2.057 (9.596)	3.701 (9.181)	–0.265* (0.131)	–0.249* (0.118)

log frequency	–88.212*** (9.943)	–93.193*** (10.627)	1.664*** (0.147)	1.668*** (0.146)

valence		–20.299* (10.013)		0.456*** (0.132)

arousal		–27.718* (11.355)		0.487*** (0.144)

concreteness		–24.448* (10.405)		0.211 (0.128)

humor		–13.877 (11.662)		0.192 (0.146)

Constant	779.558*** (17.712)	776.354*** (17.488)	2.028*** (0.185)	2.023*** (0.177)

Observations	5,554	5,554	7,510	7,510

Log Likelihood	–37,982.720	–37,960.160	–2,684.140	–2,670.626

Akaike Inf. Crit.	75,981.440	75,944.310	5,382.280	5,363.251

Bayesian Inf. Crit.	76,034.420	76,023.780	5,430.748	5,439.415


*Note:* **p < 0.05; **p < 0.01; ***p < 0.001.

#### Accuracy

Table 8 presents the results of the LME model for Accuracy. The overall mean accuracy was 98.18% (*SD* = 13.37). The following fixed effects were significant: orthographic neighborhood size, mean bigram frequency, word frequency, valence, and arousal. Two out of the four variables of interest were significant predictors of Accuracy. Specifically, positively valenced words were responded to more accurately than negatively valenced words (standardized *β* = 0.456, *SE* = 0.132, *z* = 3.44, *p* < .001), and highly arousing words were responded to more accurately than less arousing words (standardized *β* = 0.487, *SE* = 0.144, *t* = 3.38, *p* < .001). Concreteness and humor did not significantly predict accuracy. The likelihood ratio test indicated that the inclusion of lexical-semantic and affective measures significantly improved Accuracy model fit, *χ*^2^ = 27.0, *df* = 4, *p* < .001.

### LLM-Generated Norms

Valence, arousal, concreteness, and humor ratings obtained from GPT-4 were entered into the LME model in Step 2 (base+norms model) as described above, specifically, the best performing LLM measure (weighted ratings + specific prompt). This analysis would enable us to see if the LLM-generated norms would be as predictive of human linguistic performance as the human-generated norms as described above. In addition, we evaluate the Akaike information criterion (AIC) to provide a comparison across non-nested models. Generally, models that have a lower AIC score are preferred as the AIC represents the relative amount of information loss that occurred when fitting the model to the data (Wagenmakers & Farrell, 2004). Table 9 shows the results of the RT and Accuracy models where the ratings were obtained from humans were used as predictors, as well as the corresponding models where the ratings were obtained from GPT-4 were used as predictors.

**Table 9:** Comparing human-generated and LLM-generated norms.


	*DEPENDENT VARIABLE*			

	RT		ACC	
	
	*LINEAR MIXED-EFFECTS*		*GENERALIZED LINEAR MIXED-EFFECTS*	
	
	HUMAN	CHATGPT	HUMAN	CHATGPT
	
	(1)	(2)	(3)	(4)

no. of letters	52.229*** (11.246)	56.792*** (11.256)	0.041 (0.156)	0.009 (0.164)

orthographic neighborhood size	56.943*** (12.024)	58.536*** (12.161)	–0.397** (0.152)	–0.366* (0.162)

mean bigram frequency	3.701 (9.181)	–2.214 (9.470)	–0.249* (0.118)	–0.236 (0.128)

log frequency	–93.193*** (10.627)	–90.895*** (9.935)	1.668*** (0.146)	1.685*** (0.146)

valence	–20.299* (10.013)		0.456*** (0.132)	

arousal	–27.718* (11.355)		0.487*** (0.144)	

concreteness	–24.448* (10.405)		0.211 (0.128)	

humor	–13.877 (11.662)		0.192 (0.146)	

valence (gpt)		–4.719 (9.280)		0.137 (0.126)

arousal (gpt)		–10.646 (9.312)		0.245* (0.123)

concreteness (gpt)		–22.781* (9.747)		0.165 (0.129)

humor (gpt)		–32.490*** (9.469)		0.358** (0.129)

Constant	776.354*** (17.488)	777.769*** (17.509)	2.023*** (0.177)	2.031*** (0.181)

Observations	5,554	5,554	7,510	7,510

Log Likelihood	–37,960.160	–37,961.750	–2,670.626	–2,677.244

Akaike Inf. Crit.	75,944.310	75,947.500	5,363.251	5,376.488

Bayesian Inf. Crit.	76,023.780	76,026.970	5,439.415	5,452.652


*Note:* **p < 0.05; **p < 0.01; ***p < 0.001.

#### RT

The following fixed effects were significant: number of letters, orthographic neighborhood size, word frequency, concreteness, and humor. Two out of the four LLM-generated variables of interest were significant predictors of RT. Specifically, concrete words were responded to more quickly than abstract words (standardized *β* = –22.8, *SE* = 9.75, *t* = –2.34, *p* = .021), and funnier words were responded to more quickly than less funny words (standardized *β* = –32.5, *SE* = 9.47, *t* = –3.43, *p* < .001). Both valence and arousal did not significantly predict RT. A comparison of the AICs showed that the AIC of the human rating model (76003.0) was smaller than that of the LLM rating model (76005.5).

#### Accuracy

The following fixed effects were significant: orthographic neighborhood size, word frequency, arousal, and humor. Two out of the four LLM-generated variables of interest were significant predictors of Accuracy. Specifically, highly arousing words were responded to more accurately than less arousing words (standardized *β* = 0.245, *SE* = 0.123, *t* = 1.99, *p* = .047), and funnier words were responded to more quickly than less funny words (standardized *β* = 0.358, *SE* = 0.129, *t* = 2.77, *p* = .006). Both valence and concreteness did not significantly predict accuracy. A comparison of the AICs showed that the AIC of the human rating model (5363.3) was smaller than that of the LLM rating model (5376.5).

## General Discussion

In this paper, we provide lexical-semantic and affective norms of valence, arousal, concreteness, and humor, for a set of almost 300 words and concepts unique to Singapore Colloquial English. We further demonstrated their relevance for lexical processing, where words that were more positively valenced and more arousing led to faster and more accurate responses, and words that were more concrete led to faster responses.

First, it is worth noting that the findings from the visual lexical decision tasks are generally in line with the existing literature on affective and concreteness effects in lexical processing, with some exceptions. Specifically, a processing advantage was observed for words that were more positively valenced and more arousing. Although a curvilinear relationship has been reported in the literature where there is enhanced processing for emotional items relative to neutral items regardless of polarity (Kousta et al., 2009), we did not find support for such a relationship (see “Supplementary Materials” in the OSF), but this is potentially due to the limited numbers of items that were tested in the present paper. The enhanced processing of positive items relative to negative items is consistent with the automatic vigilance model of emotion (Pratto & John, 1991), where negatively-valenced stimuli automatically capture attentional resources, and also consistent with the results of an large-scale analysis of megastudy data by Kuperman et al. (2014) who reported that negative words are recognized more slowly than positive words. However, the effects of arousal found in this study, where arousing SCE words were more quickly recognized than less arousing SCE words, was inconsistent with that same analysis by Kuperman and colleagues, who found that arousing words are recognized more slowly than calming words (Kuperman et al., 2014). A processing advantage was also observed for SCE words that were more concrete. This is consistent with previous work by Goh et al. (2016) and Bottini et al. (2022) who showed a processing advantage for concrete words in lexical decision tasks. Overall, the VLDT analyses provide converging evidence that affective and semantic variables contribute important variance that explain word processing performance. At the same time it is important to note that these results are established for a small subset of items in the SCE vocabulary (relative to the megastudy approach taken by most of the studies in the literature review), and future research is necessary to confirm that these patterns are consistent over a wider range of words and concepts. To the best of our knowledge, our results also serve as a first demonstration of psycholinguistic lexical effects when processing colloquial language of a creole.

Nevertheless, it is also important to discuss why humor, unlike the other three variables, did not significantly contribute to visual lexical decision performance. Based on Engelthaler and Hills (2018)’s correlation analysis, we expected to find more humorous words to be responded to more slowly. However, the effect of human-generated humor norms was non-signficant and the effect of LLM-generated humor norms was in the opposite direction. One possibility is that humor effects may only emerge in a task that involved deeper semantic processing of the items. This is consistent with the idea that lexical processing is flexible and can be modulated by task demands (Balota & Yap, 2006). For instance, studies have found stronger semantic effects in a semantic decision task as compared to a lexical decision task, since the former invokes a deeper level of semantic processing in order to make a concrete-abstract decision (Pexman et al., 2008; Yap et al., 2011). Furthermore, the relatively low correlation between human-generated humor norms and LLM-generated humor norms relative to the other three norms suggest that the “word humor” construct may be particularly challenging for a LLM to summarize and convey in its response–a potential indicator of this can be seen in Figure 2 where the modal LLM rating for humor was 3 (neither humorous or humorless) as compared to the more normally distributed humor ratings for the human raters. Another potential reason for the low correlation is Engelthaler and Hills (2018)’s original observation that split-half reliabilities of individual humor ratings were somewhat lower than other norms, suggesting considerable individual differences in word humor ratings. All of the above suggest that humor effects may be especially difficult to detect in a task that does not involve a lot of semantic processing, particularly when there may be strong individual differences associated with word humor.

As discussed in the Introduction, current coverage of lexical-semantic and affective norms is limited to a handful of languages, and among those languages (such as English), norms are mostly constructed using data from speakers of dominant dialects of that language. One might ask why is it is important to develop norms for a different dialect of a language–wouldn’t it be more efficient and cost-effective to simply use the norms developed for a different dialect of the same language? Indeed, Siew (2024a)’s comparison of word humor norms across North American English, British English, and Singapore English (for words found in all three dialects) found them to be highly correlated with each other. Nevertheless, Siew (2024a) also reported a more subtle finding: Humor ratings were mediated by word usage statistics specific to the dialect of English spoken. Specifically, words that were more common (i.e., of higher frequency) in a corpora that represented the dialect of English spoken were rated as less humorous, suggesting that speakers of the same language are sensitive to the specific usage patterns of their own dialect, which can have downstream impacts on lexical processing (C. D. Martin et al., 2016; Van Heuven et al., 2014) and linguistic rating tasks (Siew, 2024a). Such results emphasize how assuming the existence of monolithic “English speakers” ultimately undermines the variability that exists within this inherently diverse group (Kirk, 2023), and also highlight the potential contributions that investigating less common well known varieties of languages can offer to psycholinguistics.

We argue that semantic dimensions such as valence, arousal, concreteness, and humor offer meaningful ways to study the meaning spaces of colloquial language. As demonstrated by Osgood (1964) in his ambitious comparison of affective judgments to words across 16 languages, such semantic-affective dimensions can offer tractable frames of reference for conducting cross-cultural comparisons across languages. With continued development of lexical-semantic and affective norms for SCE vocabulary, we can start to ask interesting questions about the nature of a lexicon that is shaped by communicative experience involving both the standard and informal varieties of Singapore English. For instance, are there any tendencies for colloquial language to dominate certain meaning-affective spaces in the lexicon? Recent work from our lab has shown varying levels of lexical knowledge of SCE vocabulary among younger and older speakers, raising provocative questions about the longevity and survival of Singlish in the future (Siew, 2024b)–might colloquial vocabulary that is more highly arousing, more extremely valenced, or more humorous enable its “survival” or persistence in a lexicon dominated by the standard vocabulary? Given the conflicting opinions associated with the use of Singlish in Singapore (Ching, 2020; Rubdy, 2001), how might individual language attitudes and solidarity mediate lexical processing of SCE items relative to SSE items (see Hernández-Rivera et al. (2024), for an example of how these topics have been studied among bilinguals in Montreal)? Although these are all highly speculative questions, we hope that they highlight how studying under-investigated languages and varieties enriches the kinds of questions that could be asked in psycholinguistic research, beyond what might be asked if language research were conveniently limited to speakers of a handful of the most prestigious languages (Blasi et al., 2022).

### Using LLMs to provide psycholinguistic norms for under-resourced languages

This paper also explored the possibility of using LLMs to obtain lexical-semantic and affective ratings for concepts unique to Singapore English. LLM-generated ratings obtained from GPT-4 were compared to human ratings. Results indicated that while the correlations between LLM ratings and human ratings were positive and statistically significant, there was quite a lot of variance in the LLM performance. Specifically, correlations were much higher when the prompt given included a reference to Singapore English, indicating that a higher level of prompt engineering may be necessary to “nudge” LLMs into an appropriate context prior to generating ratings for under-resourced and under-represented languages and dialects. Correlations were also highest for valence, and lowest for humor, which further indicate that LLMs may provide better or worse results depending on the type of lexical information that is being queried. Despite the significant correlations, it is worth highlighting that the overall distributions of the ratings were sigificantly different across human raters and the LLM (see Figure 2).

Given the previous work showed that GPT-4 was able to generate valid ratings for Spanish, a language that the model was not specifically trained on (Z. Li et al., 2024; Martínez, Conde, et al., 2024), the results for Singapore English could be considered somewhat disappointing in comparison. However, this might not be too surprising because even if one does not have access to the raw contents of the training data, one could still reasonably assume that Spanish text, while not forming the majority of the LLM’s training data, is several times larger than the amount of English text produced by native speakers of Singapore English. Indeed, computational experiments have suggested strong correlations between the LLM’s performance in various languages and the proportion of those languages in its pre-training corpus (Z. Li et al., 2024). Our results may serve as a gentle warning that LLMs are not a panacea for psycholinguistic research, particularly when trying use them to develop lexical norms for minority languages and dialects. It is important to develop and validate LLM-generated norms in close conjunction with data that is ideally newly obtained from humans to guard against data leakage and contamination (Trott, 2024).

Finally, we compared the ability of LLM-generated norms to account for human performance on the VLDT. As the results in Part 3 showed, the model that used human ratings as predictors out-performed the model that used GPT ratings as predictors on the AIC model performance measure. In addition, when GPT ratings were used as predictors instead of the human ratings, the overall patterns were different from the original model. Specifically, the human model showed consistent effects of valence and arousal across both RT and accuracy, whereas the only consistent effect across RT and accuracy in the GPT model was humor, which was not originally significant in the human model. This is a somewhat surprising finding given that the correlations between the human and GPT ratings were much higher for valence (*r* = 0.78) and arousal (*r* = 0.59) than for humor (*r* = 0.39). While it is difficult to provide any convincing explanation for this discrepancy, these results provide an initial argument that LLM-generated norms may need to be held to a higher standard. Beyond establishing a high correlation with human generated norms or annotations (typically done in previous studies, e.g., (Martínez, Molero, et al., 2024; Trott, 2024)), researchers should also assess if LLM-generated norms are able to account for known behavioral effects in psycholinguistic experiments to a better extent than human norms, and discrepancies in the behavioral patterns and overall distribution patterns such as those reported here could provide important clues into potential limitations of LLM-generated norms. After all, a key goal of developing lexical-semantic and affective norms is to enable a better characterization of empirical data in psycholinguistics–this is why researchers continue to develop better and bigger word frequency databases (Brysbaert & New, 2009) or propose new variables such as prevalence or word knowledge (Keuleers et al., 2015) based on their improved performance at accounting for variance in large-scale behavioral datasets.

### Limitations and future directions

A clear limitation of this research is that we have only collected lexical-norms for a small subset of the SCE vocabulary, which pales in comparison to the sizes of modern psycholinguistic databases (consisting of at least several thousand items). However, because lexical-semantic and affective measures have never been developed for concepts unique to SCE, a pragmatic approach that we have decided on was to first obtain norms for a subset of well-known items to determine if meaningful ratings could be obtained. Our results clearly show that speakers of Singapore English converge on their ratings of these concepts, and that these ratings have implications for lexical processing. The current results provide us with some confidence that it is a worthwhile research pursuit to persist with our data collection efforts for the rest of our database. They also motivate us to potentially extend data collection to other types of norms, such as prevalence, which provides an index of word knowledge across the population (Brysbaert et al., 2019) and a potentially important complement to word frequency information, which may be less relevant for colloquial lexical items that are more often spoken and less often written, and sensorimotor norms, which provides information about perceptual and action strength and can enhance our understanding of the sensorimotor basis of semantic representations of Singapore English concepts (Lynott et al., 2020).

Another limitation of this research is that only basic demographic varibles were collected from the participants. However, future work could collect additional information such as personality or sociolinguistic attitudes towards Singapore English, to explore how individual differences may impact elicitation of ratings on various lexical-semantic and affective dimensions, and in particular for word humor ratings which may be especially liable to well-established individual differences in humor styles (R. A. Martin et al., 2003). It is also worth mentioning that Chinese participants were over-represented in our rating sample (~89% of participants for which we had ethnicity information on), which is higher than the national proportion of 75% Chinese ethnicity (with 14% Malay, 9% Indian, and the remainder as Others). This could have an impact on the rating results depending on the item’s etymology and relative differences in usage patterns of specific items across ethnicities. Future studies should look into this issue in more detail.

In conclusion, we hope that this paper inspires researchers to devote some effort toward developing psycholinguistic norms for informal, colloquial languages (such as creoles) and other less studied varieties of English, and motivates researchers of Singapore English to exploit this lexical-semantic and affective database for their own research.

## Data Accessibility Statement

All norms, data, and analysis scripts can be found at https://osf.io/bjxfa.

## References

[1] Balota, D. A., & Yap, M. J. (2006). Attentional control and the flexible lexical processor: Explorations of the magic moment of word recognition. In S. Andrews (Ed.), From Inkmarks to Ideas: Current Issues in Lexical Processing (pp. 229–258).

[2] Balota, D. A., Yap, M. J., Hutchison, K. A., Cortese, M. J., Kessler, B., Loftis, B., Neely, J. H., Nelson, D. L., Simpson, G. B., & Treiman, R. (2007). The English lexicon project. Behavior Research Methods, 39(3), 445–459. http://www.springerlink.com/index/l7121702t08h0456.pdf 17958156 10.3758/bf03193014

[3] Bartko, J. J. (1966). The Intraclass Correlation Coefficient as a Measure of Reliability. Psychological Reports, 19(1), 3–11. 10.2466/pr0.1966.19.1.3 5942109

[4] Bates, D., Mächler, M., Bolker, B., & Walker, S. (2014). Fitting Linear Mixed-Effects Models using Lme4. 10.18637/jss.v067.i01

[5] Blasi, D. E., Henrich, J., Adamou, E., Kemmerer, D., & Majid, A. (2022). Over-reliance on English hinders cognitive science. Trends in Cognitive Sciences, 26(12), 1153–1170. 10.1016/j.tics.2022.09.015 36253221

[6] Bolton, K., Botha, W., & Kirkpatrick, A. (Eds.). (2020). The handbook of Asian Englishes. Wiley Blackwell. 10.1002/9781118791882

[7] Bottini, R., Morucci, P., D’Urso, A., Collignon, O., & Crepaldi, D. (2022). The concreteness advantage in lexical decision does not depend on perceptual simulations. Journal of Experimental Psychology: General, 151(3), 731–738. 10.1037/xge0001090 34498912

[8] Brysbaert, M., & Diependaele, K. (2013). Dealing with zero word frequencies: A review of the existing rules of thumb and a suggestion for an evidence-based choice. Behavior Research Methods, 45(2), 422–430. 10.3758/s13428-012-0270-5 23055175

[9] Brysbaert, M., Mandera, P., McCormick, S. F., & Keuleers, E. (2019). Word prevalence norms for 62,000 English lemmas. Behavior Research Methods, 51(2), 467–479. 10.3758/s13428-018-1077-9 29967979

[10] Brysbaert, M., & New, B. (2009). Moving beyond Kučera and Francis: A critical evaluation of current word frequency norms and the introduction of a new and improved word frequency measure for American English. Behavior Research Methods, 41(4), 977–990. http://link.springer.com/article/10.3758/BRM.41.4.977 19897807 10.3758/BRM.41.4.977

[11] Brysbaert, M., Stevens, M., De Deyne, S., Voorspoels, W., & Storms, G. (2014). Norms of age of acquisition and concreteness for 30,000 Dutch words. Acta Psychologica, 150, 80–84. 10.1016/j.actpsy.2014.04.010 24831463

[12] Brysbaert, M., Warriner, A. B., & Kuperman, V. (2014). Concreteness ratings for 40 thousand generally known English word lemmas. Behavior Research Methods, 46(3), 904–911. 10.3758/s13428-013-0403-5 24142837

[13] Buchanan, E. M., Valentine, K. D., & Maxwell, N. P. (2019). LAB: Linguistic Annotated Bibliography – a searchable portal for normed database information. Behavior Research Methods, 51(4), 1878–1888. 10.3758/s13428-018-1130-8 30284211

[14] Cavallaro, F., Ng, B. C., & Tan, Y. Y. (2020). Singapore English. In K. Bolton, W. Botha, & A. Kirkpatrick (Eds.), The Handbook of Asian Englishes. John Wiley & Sons. 10.1002/9781118791882.ch18

[15] Ching, W. S. (2020). Does Singlish Contribute to Singaporean’s National Identity, and do Singaporeans Support Formal Recognition of it? International Journal of Academic Research in Progressive Education and Development, 9(2), 96–112. 10.6007/IJARPED/v9-i2/7244

[16] Diveica, V., Muraki, E. J., Binney, R. J., & Pexman, P. M. (2024). Socialness effects in lexical–semantic processing. Journal of Experimental Psychology: Learning, Memory, and Cognition, 50(8), 1329–1343. 10.1037/xlm0001328 38512176

[17] Diveica, V., Pexman, P. M., & Binney, R. J. (2022). Quantifying social semantics: An inclusive definition of socialness and ratings for 8388 English words. Behavior Research Methods, 55(2), 461–473. 10.3758/s13428-022-01810-x 35286618 PMC10027635

[18] Duan, X., Li, S., & Cai, Z. G. (2024). MacBehaviour: An R package for behavioural experimentation on large language models. Behavior Research Methods, 57(1), 19. 10.3758/s13428-024-02524-y 39694977 PMC11655609

[19] Engelthaler, T., & Hills, T. T. (2018). Humor norms for 4,997 English words. Behavior Research Methods, 50(3), 1116–1124. 10.3758/s13428-017-0930-6 28710716 PMC5990549

[20] Fatima, A., Li, Y., Hills, T. T., & Stella, M. (2021). DASentimental: Detecting Depression, Anxiety, and Stress in Texts via Emotional Recall, Cognitive Networks, and Machine Learning. Big Data and Cognitive Computing, 5(4), 77. 10.3390/bdcc5040077

[21] Goh, W. D., Yap, M. J., & Chee, Q. W. (2020). The Auditory English Lexicon Project: A multi-talker, multi-region psycholinguistic database of 10,170 spoken words and nonwords. Behavior Research Methods. 10.3758/s13428-020-01352-0 32291734

[22] Goh, W. D., Yap, M. J., Lau, M. C., Ng, M. M. R., & Tan, L.-C. (2016). Semantic Richness Effects in Spoken Word Recognition: A Lexical Decision and Semantic Categorization Megastudy. Frontiers in Psychology, 7. 10.3389/fpsyg.2016.00976 PMC4923159 27445936

[23] Gonzales, W. D. W., Hiramoto, M., Leimgruber, J. R. E., & Lim, J. J. (2023). The Corpus of Singapore English Messages (CoSEM). World Englishes, 42(2), 371–388. 10.1111/weng.12534

[24] Groot, A. M. B. D., & Keijzer, R. (2000). What Is Hard to Learn Is Easy to Forget: The Roles of Word Concreteness, Cognate Status, and Word Frequency in Foreign-Language Vocabulary Learning and Forgetting. Language Learning, 50(1), 1–56. 10.1111/0023-8333.00110

[25] Gupta, A. F. (1994). The step-tongue: Children’s English in Singapore. Multilingual Matters.

[26] Haro, J., Hinojosa, J. A., & Ferré, P. (2024). The role of individual differences in emotional word recognition: Insights from a large-scale lexical decision study. Behavior Research Methods, 56(8), 8501–8520. 10.3758/s13428-024-02488-z 39231911 PMC11525433

[27] Hernández-Rivera, E., Kalogeris, A., Tiv, M., & Titone, D. (2024). Self-evaluations and the language of the beholder: Objective performance and language solidarity predict L2 and L1 self-evaluations in bilingual adults. Cognitive Research: Principles and Implications, 9(1), 75. 10.1186/s41235-024-00592-4 39495425 PMC11535130

[28] Hills, T. T., & Adelman, J. S. (2015). Recent evolution of learnability in American English from 1800 to 2000. Cognition, 143, 87–92. 10.1016/j.cognition.2015.06.009 26117487

[29] Ho, D., Hamzah, D., Poria, S., & Cambria, E. (2018). Singlish SenticNet: A Concept-Based Sentiment Resource for Singapore English. 2018 IEEE Symposium Series on Computational Intelligence (SSCI), 1285–1291. 10.1109/SSCI.2018.8628796

[30] Joser, J. R., Dedace, J. R., Daclizon, K., Salvaña, P. L., Nacionales, J., & Claridad, N. (2023). The rhetoric of comedy: Exploring the language and humor styles of Filipino stand-up comedians. Journal of Language and Pragmatics Studies, 2(2), 71–88. 10.58881/jlps.v2i2.17

[31] Keuleers, E., Stevens, M., Mandera, P., & Brysbaert, M. (2015). Word knowledge in the crowd: Measuring vocabulary size and word prevalence in a massive online experiment. The Quarterly Journal of Experimental Psychology, 68(8), 1665–1692. 10.1080/17470218.2015.1022560 25715025

[32] Kidd, E., & Garcia, R. (2022). How diverse is child language acquisition research? First Language, 42(6), 703–735. 10.1177/01427237211066405 PMC9605524 36310838

[33] Kirk, N. W. (2023). MIND your language(s): Recognizing Minority, Indigenous, Non-standard(ized), and Dialect variety usage in “monolinguals.” Applied Psycholinguistics, 44(3), 358–364. 10.1017/S0142716422000467

[34] Koh, J. X., Mislan, A., Khoo, K., Ang, B., Ang, W., Ng, C., & Tan, Y. Y. (2019). Building the singapore english national speech corpus. Malay, 20(25.0), 19–13. 10.21437/Interspeech.2019-1525

[35] Kousta, S.-T., Vinson, D. P., & Vigliocco, G. (2009). Emotion words, regardless of polarity, have a processing advantage over neutral words. Cognition, 112(3), 473–481. 10.1016/j.cognition.2009.06.007 19591976

[36] Kuperman, V., Estes, Z., Brysbaert, M., & Warriner, A. B. (2014). Emotion and language: Valence and arousal affect word recognition. Journal of Experimental Psychology: General, 143(3), 1065. 10.1037/a0035669 24490848 PMC4038659

[37] Kuznetsova, A., Brockhoff, P. B., & Christensen, R. H. B. (2017). lmerTest package: Tests in linear mixed effects models. Journal of Statistical Software, 82(13). 10.18637/jss.v082.i13

[38] Levisen, C. (2019). Biases we live by: Anglocentrism in linguistics and cognitive sciences. Language Sciences, 76, 101173. 10.1016/j.langsci.2018.05.010

[39] Li, Y., Breithaupt, F., Hills, T., Lin, Z., Chen, Y., Siew, C. S. Q., & Hertwig, R. (2024). How cognitive selection affects language change. Proceedings of the National Academy of Sciences, 121(1), e2220898120. 10.1073/pnas.2220898120 PMC10769849 38150495

[40] Li, Z., Shi, Y., Liu, Z., Yang, F., Payani, A., Liu, N., & Du, M. (2024, December 11). Language Ranker: A Metric for Quantifying LLM Performance Across High and Low-Resource Languages. 10.48550/arXiv.2404.11553

[41] Lo, P.-C., & Lim, E.-P. (2018). On Learning Psycholinguistics Tools for English-based Creole Languages using Social Media Data. 2018 IEEE International Conference on Big Data (Big Data), 751–760. 10.1109/BigData.2018.8622010

[42] Lynott, D., Connell, L., Brysbaert, M., Brand, J., & Carney, J. (2020). The Lancaster Sensorimotor Norms: Multidimensional measures of perceptual and action strength for 40,000 English words. Behavior Research Methods, 52(3), 1271–1291. 10.3758/s13428-019-01316-z 31832879 PMC7280349

[43] Martin, C. D., Garcia, X., Potter, D., Melinger, A., & Costa, A. (2016). *Holiday* or *Vacation*? The processing of variation in vocabulary across dialects. Language, Cognition and Neuroscience, 31(3), 375–390. 10.1080/23273798.2015.1100750

[44] Martin, R. A., Puhlik-Doris, P., Larsen, G., Gray, J., & Weir, K. (2003). Individual differences in uses of humor and their relation to psychological well-being: Development of the Humor Styles Questionnaire. Journal of Research in Personality, 37(1), 48–75. 10.1016/S0092-6566(02)00534-2

[45] Martínez, G., Conde, J., Reviriego, P., & Brysbaert, M. (2024). AI-generated estimates of familiarity, concreteness, valence, and arousal for over 100,000 Spanish words. Quarterly Journal of Experimental Psychology, 17470218241306694. 10.1177/17470218241306694 39614682

[46] Martínez, G., Molero, J. D., González, S., Conde, J., Brysbaert, M., & Reviriego, P. (2024). Using large language models to estimate features of multi-word expressions: Concreteness, valence, arousal. Behavior Research Methods, 57(1), 5. 10.3758/s13428-024-02515-z 39633225

[47] Mohammad, S. M., & Turney, P. D. (2013). CROWDSOURCING A WORD–EMOTION ASSOCIATION LEXICON. Computational Intelligence, 29(3), 436–465. 10.1111/j.1467-8640.2012.00460.x

[48] New, B., Bourgin, J., Barra, J., & Pallier, C. (2024). UniPseudo: A universal pseudoword generator. Quarterly Journal of Experimental Psychology, 77(2), 278–286. 10.1177/17470218231164373 36891822

[49] Osgood, C. E. (1964). Semantic Differential Technique in the Comparative Study of Cultures. American Anthropologist, 66(3), 171–200. http://www.jstor.org/stable/669329

[50] Osgood, C. E., Suci, G. J., & Tannenbaum, P. H. (1957). The measurement of meaning. University of Illinois press.

[51] Paivio, A. (2013). Dual coding theory, word abstractness, and emotion: A critical review of Kousta et al. (2011). Journal of Experimental Psychology: General, 142(1), 282–287. 10.1037/a0027004 23398183

[52] Pexman, P. M., Hargreaves, I. S., Siakaluk, P. D., Bodner, G. E., & Pope, J. (2008). There are many ways to be rich: Effects of three measures of semantic richness on visual word recognition. Psychonomic Bulletin & Review, 15(1), 161–167. 10.3758/PBR.15.1.161 18605497

[53] Pratto, F., & John, O. P. (1991). Automatic vigilance: The attention-grabbing power of negative social information. Journal of Personality and Social Psychology, 61(3), 380–391. 10.1037/0022-3514.61.3.380 1941510

[54] Rubdy, R. (2001). Creative destruction: Singapore’s Speak Good English movement. World Englishes, 20(3), 341–355. 10.1111/1467-971X.00219

[55] Ruiz-Gurillo, L. (2021). Disrupted vs. Sustained humor in colloquial conversations in peninsular Spanish. Journal of Pragmatics, 178, 162–174. 10.1016/j.pragma.2021.03.011

[56] Schwanenflugel, P. J., Harnishfeger, K. K., & Stowe, R. W. (1988). Context availability and lexical decisions for abstract and concrete words. Journal of Memory and Language, 27(5), 499–520. 10.1016/0749-596X(88)90022-8

[57] Shrout, P. E., & Fleiss, J. L. (1979). Intraclass correlations: Uses in assessing rater reliability. Psychological Bulletin, 86(2), 420–428. 10.1037/0033-2909.86.2.420 18839484

[58] Siew, C. S. Q. (2024a). A comparison of word humor ratings across speakers of North American, British, and Singapore English. Memory & Cognition. 10.3758/s13421-024-01587-8 38865076

[59] Siew, C. S. Q. (2024b). Tracking Lexical Knowledge of Concepts Unique to Singapore English Among Speakers of Singapore English. Proceedings of the 46th Annual Meeting of the Cognitive Science Society, 46. https://escholarship.org/uc/item/8d8595gm

[60] Siew, C. S. Q., Engelthaler, T., & Hills, T. T. (2022). Nymph piss and gravy orgies: Local and global contrast effects in relational humor. Journal of Experimental Psychology: Learning, Memory, and Cognition, 48(7), 1047–1063. 10.1037/xlm0001120 35404646

[61] Singh, L., Cristia, A., Karasik, L. B., Rajendra, S. J., & Oakes, L. M. (2023). Diversity and representation in infant research: Barriers and bridges toward a globalized science of infant development. Infancy, 28(4), 708–737. 10.1111/infa.12545 37211974

[62] Stadthagen-Gonzalez, H., Imbault, C., Pérez Sánchez, M. A., & Brysbaert, M. (2017). Norms of valence and arousal for 14,031 Spanish words. Behavior Research Methods, 49(1), 111–123. 10.3758/s13428-015-0700-2 26850056

[63] Tan, Y.-Y. (2017). Singlish: An illegitimate conception in Singapore’s language policies? European Journal of Language Policy, 9(1), 85–104. 10.3828/ejlp.2017.6

[64] Taylor, J. E., Rousselet, G. A., Scheepers, C., & Sereno, S. C. (2022). Rating norms should be calculated from cumulative link mixed effects models. Behavior Research Methods, 55(5), 2175–2196. 10.3758/s13428-022-01814-7 36103049 PMC10439063

[65] Teixeira, A. S., Talaga, S., Swanson, T. J., & Stella, M. (2021). Revealing semantic and emotional structure of suicide notes with cognitive network science. Scientific Reports, 11(1, 1), 19423. 10.1038/s41598-021-98147-w 34593826 PMC8484592

[66] Thalmayer, A. G., Toscanelli, C., & Arnett, J. J. (2021). The neglected 95% revisited: Is American psychology becoming less American? American Psychologist, 76(1), 116–129. 10.1037/amp0000622 32271027

[67] Trott, S. (2024). Can large language models help augment English psycholinguistic datasets? Behavior Research Methods, 56(6), 6082–6100. 10.3758/s13428-024-02337-z 38261264 PMC11335796

[68] Van Heuven, W. J. B., Mandera, P., Keuleers, E., & Brysbaert, M. (2014). Subtlex-UK: A New and Improved Word Frequency Database for British English. Quarterly Journal of Experimental Psychology, 67(6), 1176–1190. 10.1080/17470218.2013.850521 24417251

[69] Wagenmakers, E.-J., & Farrell, S. (2004). AIC model selection using Akaike weights. Psychonomic Bulletin & Review, 11(1), 192–196. 10.3758/BF03206482 15117008

[70] Warriner, A. B., Kuperman, V., & Brysbaert, M. (2013). Norms of valence, arousal, and dominance for 13,915 English lemmas. Behavior Research Methods, 45(4), 1191–1207. 10.3758/s13428-012-0314-x 23404613

[71] Westbury, C., & Hollis, G. (2019). Wriggly, squiffy, lummox, and boobs: What makes some words funny? Journal of Experimental Psychology: General, 148(1), 97–123. 10.1037/xge0000467 30335445

[72] Westbury, C., Shaoul, C., Moroschan, G., & Ramscar, M. (2016). Telling the world’s least funny jokes: On the quantification of humor as entropy. Journal of Memory and Language, 86, 141–156. 10.1016/j.jml.2015.09.001

[73] Wong, J. J., & Siew, C. S. Q. (2024). Preliminary Data from the Small World of Singlish Words Project: Examining Responses to Common Singlish Words. Journal of Open Psychology Data, 12(1), 3. 10.5334/jopd.108 40687675 PMC12270029

[74] Yap, M. J., Tan, S. E., Pexman, P. M., & Hargreaves, I. S. (2011). Is more always better? Effects of semantic richness on lexical decision, speeded pronunciation, and semantic classification. Psychonomic Bulletin & Review, 18(4), 742–750. 10.3758/s13423-011-0092-y 21494916

